# A model-independent redundancy measure for human *versus* ChatGPT authorship discrimination using a Bayesian probabilistic approach

**DOI:** 10.1038/s41598-023-46390-8

**Published:** 2023-11-06

**Authors:** Silvia Bozza, Claude-Alain Roten, Antoine Jover, Valentina Cammarota, Lionel Pousaz, Franco Taroni

**Affiliations:** 1 Ca’ Foscari University of Venice, Department of Economics, Venice, 30121 Italy; 2 OrphAnalytics SA, Vevey, 1800 Switzerland; 3 University of Lausanne, School of Criminal Justice, Lausanne, 1015 Switzerland

**Keywords:** Computational science, Scientific data, Statistics

## Abstract

The academic and scientific world in general is increasingly concerned about their inability to determine and ascertain the identity of the writer of a text. More and more often the question arises as to whether a scientific article or work handed in by a student was actually produced by the alleged author of the questioned text. The role of artificial intelligence (AI) is increasingly debated due to its dangers of undeclared use. A current example is undoubtedly the undeclared use of ChatGPT to write a scientific text. The article promotes an AI model-independent redundancy measure to support discrimination between hypotheses on authorship of various multilingual texts written by humans or produced by intelligence media such as ChatGPT. The syntax of texts written by humans tends to differ from that of texts produced by AIs. This difference can be grasped and quantified even with short texts (i.e. 1800 characters). This aspect of length is extremely important, because short texts imply a greater difficulty of analysis to characterize authorship. To meet the efficiency criteria required for the evaluation of forensic evidence, a probabilistic approach is implemented. In particular, to assess the value of the redundancy measure and to offer a consistent classification criterion, a metric called Bayes factor is implemented. The proposed Bayesian probabilistic method represents an original approach in stylometry. Analyses performed over multilingual texts (English and French) covering different scientific and human areas of interest (forensic science and socio-psycho-artistic topics) reveal the feasibility of a successful authorship discrimination with limited misclassification rates. Model performance is satisfactory even with small sample sizes.

## Introduction

The controversy over the authorship of texts published not only in scientific journals but also in publications ranging from legal or social to psychological themes is increasingly topical. The academic world, too, is on the alert because it seems to find itself lacking a valid support for investigating the authorship of texts potentially drafted with the help of what many now call ‘artificial intelligence’ (AI). One example, above all, is the use of ChatGPT as a support or as a full substitute for text editing. The judicial community is also alarmed with reference to sided copyright issues. The Swiss legal (online) journal *Jusletter*, regularly announces series of special conferences on this topic (www.weblab.ch). In fact, at least since the first lawsuits were filed by authors against AI application operators in the United States, the question has become stronger as to what copyright implications the use of AI in general and ChatGPT in particular have. Description of the first US lawsuits can be found in:

- Kyle Wiggers, The current legal cases against generative AI are just the beginning. January 27, 2023 at www.techcrunch.com;
- Blake Brittain, AI-created images lose U.S. copyrights in test for new technology. February 23, 2023 at www.reuters.com;
- Tiana Loving, Current AI copyright cases - The unauthorized use of copyrighted material as training data. March 30, 2023 at www.copyrightalliance.org;
- Blake Brittain, Lawsuit says OpenAI violated US authors’ copyrights to train AI chatbot. June 29, 2023 at www.reuters.com;
- Ella Creamer, Authors file a lawsuit against OpenAI for unlawfully ‘ingesting’ their book. July 5, 2023 at www.theguardian.com.

The question of main interest is whether it is possible to discriminate - or at least highlight a trend on - a text authored by a human (e.g., a scientist) from a text, on the same scientific or social subject, delivered by ChatGPT regardless of the model used by this AI media to reproduce human writing. This challenge is reminiscent of a series of scientific events on digital text analysis held in 2019, e.g.^1–3^ where identifying tweets written by humans or bots with malicious intent was one of the objectives. Vocabulary richness, defined as vocabulary amplitude^4^, is one of the most well-known markers of lexical features that can aid stylistic analysis. Style implies a set of quantifiable characteristics^5,6^, determined by the syntax of a text, specific to each person^7^ and vocabulary richness seemed to play an important role in distinguishing between machine’s and human’s writings^8^. But this is not the end of the story just because ChatGPT can be instructed to write by applying a specially defined style. This makes the specificity of ChatGPT’s style non-existent or at least difficult to be discriminated from that of a specific category of human beings.

In this case, *N*-grams (sequences of *N* words including punctuation) have been used to characterize a measure of singularity related to redundancy phenomena appearing in a written text independently of the model (algorithm) used to produce it. In this respect, the syntax in texts authored by humans tends to differ from those characterizing texts produced by the artificial intelligence which is less richer in vocabulary.

A probabilistic approach for the evaluation of stylometric data is implemented with the aim of discriminating between classes of putative authors (i.e., Human versus ChatGPT) in total respect of the efficiency criteria that characterize the evaluation of scientific evidence in a forensic and judicial context^9,10^ and^11^.

The paper is structured as follows. In the section ‘Materials and methods’ there are described the available text materials characterizing the populations of interest (Human and ChatGPT) and the stylometry measure used for the extraction of data from available texts. It is also illustrated the use of the Bayes factor as a measure for evidence evaluation and it is briefly presented the statistical model that is applied. Results of performed analyses are presented in section ‘Results’, where it is shown that the proposed probabilistic approach may offer a valuable contribution to help tackling the question of authorship. Section ‘Discussion and conclusion’, finally, concludes the paper.

## Material and methods

### Available material

Forensic science is a scientific area of judicial and social interest. Our attention was mainly directed towards this field because of its impact on society and related judgments. Forensic practice is routinely confronted with a limited amount of trace material and comparative reference samples. Forensic scientists must be able to juggle this additional constraint. For this reason, we considered 75 articles issued from the peer-review journal *Forensic Science International* in the period 1978-1985. The choice of this time interval was guided by the need to exclude articles even only potentially written with the aid of intelligent media. Collected material is thus composed of introductory texts on forensic topics ranging from toxicology, forensic medicine, search for, and chemical characterization of, textile fibers to various accident statistics.

Starting from subjects characterizing the selected articles, ChatGPT (ChatGPT Mar 14 Version, available at https://chat.openai.com/chat) was explicitly asked to draft scientific texts addressing such topics in an extension that could characterize the introductory part of a scientific article. Without precise specification, the length of the texts delivered by ChatGPT varies between 800 and 2000 characters. The collected drafts have been abruptly cut at 1800 characters to ease their comparative standardization (a normalizing approach on text lengths has also been implemented with extremely close results). A total number of 37 and 57 texts, respectively, were retained among those collected from the peer-review journal (*Human*) and those produced by the artificial intelligence (*ChatGPT *). A population of 94 texts of 1800 characters’ length is therefore available. It is worth noting that this is well below the length of the present article. This aspect of length is extremely important. Indeed, short texts imply an extended difficulty of analysis in order to characterize authorship.

In addition, a second set of 71 original texts authored by Master’s students of a Swiss University Arts Faculty in a pre-ChatGPT period were used and compared with 49 texts produced by ChatGPT on the same selected topics, ranging from the role of Renaissance painting in the conception of beauty to the role of social media on the behaviour of people inclined to spread their political or sexual views, or from urban development in modern towns to the difference in various cultural conceptions of the term ‘hero’. The ChatGPT texts have been generated using the following prompt: ‘Ignore all instructions before this one. You are a [role]. You have been writing [domain] essay for 10 years. Your task is now to explain the [questioned theme].’

### Redundancy measure

Sequences of *N*-grams have been summarized by a singularity measure quantifying the single appearance of a given *N*-gram (notably uni-, bi-, tri- and quadri-grams) in the questioned text. Stylometric analyses based on the occurrences of observation of selected *N*-grams have been performed using the software PATOA, a software developed by the company OrphAnalytics SA (see, www.orphanalytics.com for more information).

The style marker is quantified through singularity or redundancy measures on words. The redundancy value simply represents the complement of the singularity value; this value denotes the proportion of repetitions (at least 2) of specific *N*-grams in a given text. An absence, or at least a limitation, of redundancy in *N*-grams supports the idea that a rich and extensive vocabulary is adopted.

Note that the redundancy measure does not require any extended data set for training and acquisition of knowledge; this measure is AI model-independent, so the use of larger sample sizes does not play a fundamental role for discrimination purposes. The analysis of a case involving a small sample size is described in section ‘Results’.

In this paper, the use of *N*-grams is originally coupled with the redundancy measure. This measure allows one to characterize a document by means of the systematic identification of a pattern used within and between words and sentences, respectively. It must be emphasized that Large Language Models (LLMs) are often recommended for their alleged ability to detect the implementation of AI in text writing. There are two strategies for such a suspicious detection. On one side, most detectors use a supervised approach, taking advantage of knowledge of the LLM. On the other side, unsupervised detection of AI style without knowledge of the LLM can be implemented. This makes it possible to detect the style of AI-generated texts, which are characterized by more predictable structures, and in particular by a greater number of repetitions (e.g. of words, punctuation marks), and so by higher redundancy. Such unsupervised stylistic detection approach can be extended to multilingual contexts by virtue of the independence of the redundancy measure with respect to the algorithms that allow ChatGPT to form complete sentences.

**Figure 1:**
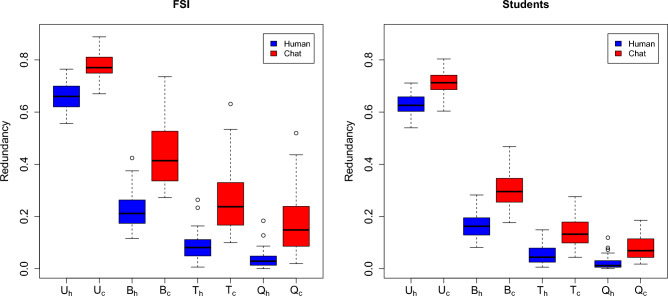
Redundancy measure for words uni- (U), bi- (B), tri- (T) and quadri- (Q) grams in the two populations: forensic science papers (FSI, left) and student’s manuscripts (Students, right). A distinction is made for texts written by humans (h), either forensic scientists or students (blue colored boxplots), and text delivered by the artificial intelligence (c), either for scientific papers or students’ texts (red colored boxplots). The first population (FSI) is characterized by texts written in English, while the second one (Students) is characterized by texts written in French.

The stylometric profile of texts of known authors allows one to rule quantitatively on such texts to tackle the problem of authorship and characterize populations. Available measurements on collected texts are presented in Fig. 1. Note that currently available detection approaches refer to algorithms for AI-generated texts based on the way a given text is generated using probable words. Measures for detection are therefore (and contrary to the redundancy measure) model-dependent.

### Bayesian probabilistic model

The style marker can be used in association with a probabilistic approach to assess its contribution for supporting authorship hypothesis, as requested in forensic science when, e.g. DNA profiles respectively related to a recovered stain and to a person of interest (i.e. a victim or a suspect) are obtained through genetical laboratory analysis and should be evaluated in the light of competing hypotheses put forward by mandating authorities representing those aspects a Court of justice seeks to reach a judgement^12^. A questioned authorship represents the key issue for a Court. Denote, for sake of simplicity, by letters $H_1$ and $H_2$ the hypotheses of interest, say $H_1$, the author of a given questioned document is a human individual, and $H_2$, the author of a given questioned document is ChatGPT, and denote by *y* the redundancy measure, also called the evidence. This problem of discrimination is treated as a problem of testing statistical hypotheses about authorship of a questioned document. Evaluation of evidence is achieved through the assignment of a Bayes factor (BF), which provides the forensic scientist with a coherent measure of the degree to which the evidence can discriminate between the different hypotheses advocated by the opposing parties at trial^13–15^:

1 $$\begin{aligned} {\textrm{BF}}= \frac{f(y\mid H_1)}{f(y\mid H_2)}. \end{aligned}$$

Bayes factor value is non-negative with no upper bound. A value greater than one provides support for the hypothesis $H_1$ (over $H_2$), and a value lower than one favors the alternative hypothesis $H_2$ (over $H_1$). Evidence for which the value is equal to 1 is neutral in that the evidence does not discriminate between the two hypotheses of interest. Although the use of Bayes factor in forensic science is a widely used approach, its application in stylometry is still unexplored.

After opportune mathematical transformation, measurements exhibit enough regularity for standard Normal parametric models to be used, $f(y\mid \theta ,\sigma ^2)=\textrm{N}(\theta ,\sigma ^2)$. A conjugate Normal-inverse-Gamma distribution $f(\theta \mid \sigma ^2)f(\sigma ^2)$ is fitted for population mean and variance, $(\theta ,\sigma ^2)$, where $f(\theta \mid \sigma ^2)=\textrm{N}(\mu ,\sigma ^2/n_0)$ and $f(\sigma ^2)=\textrm{IG}(\alpha ,\beta )$. The marginal likelihoods at the numerator and denominator of the Bayes factor in (1) can be obtained analytically,

$$\begin{aligned} f(y\mid H)=\int f(y\mid \theta ,\sigma ^2)f(\theta ,\sigma ^2)d(\theta ,\sigma ^2). \end{aligned}$$

It can be proved that $f(y\mid H)$ is a Student-t distribution centered at the prior mean $\mu$ with spread parameter $s=\frac{n_0n}{n_0+n}\alpha \beta ^{-1}$ and $2\alpha$ degrees of freedom, $\textrm{St}(\mu ,s,2\alpha )$^16^.

It might be of interest to retain all available *N*-grams and test the global support offered to competing hypotheses whenever jointly considered. The previous statistical model can be extended accordingly to handle multivariate data, as the (multivariate) Normal distribution shows a good fit to the available measurements. The prior choice falls now into the conjugate Normal-inverse-Wishart prior distribution. The marginal likelihood can again be obtained analytically and turns out to be a multivariate Student-t distribution^16^.

Data treatment, visualization and probabilistic evaluation were all carried out in the R statistical software package available at https://www.r-project.org.

## Results

To study the distribution of the Bayes factor values obtained using texts of known source (either Human or ChatGPT) selected from the available material, a leave-one-out method has been used, while the remaining data have been used to elicit model parameters. To test hypothesis $H_1$ (the putative source is human), a Bayes factor has been calculated for measurements originating from each text in the *Human* database (either FSI or Students). Analogously, to test hypothesis $H_2$ (the putative source is ChatGPT), a Bayes factor has been calculated for measurements originating from each text in the *ChatGPT* database (either for FSI or Students generated texts). All *N*-grams have been analyzed either separately, or jointly, by means of univariate and multivariate models. The best performances have been achieved whenever all *N*-grams are retained. In Table 1 there are reported BF values for cases where bi-grams (B) or all *N*-grams (M) are considered. There are also summarized the total number of cases where BF values support the correct hypothesis, that is a $BF>1$ ($BF<1$) whenever $H_1$ ($H_2$) is true, as well as the total number of cases where the wrong hypothesis is supported.

**Table 1:** Assessment of the performances of the probabilistic approach for authorship discrimination when the putative source is human ($H_1$), and when the putative source is ChatGPT ($H_2$). Analyses results are reported for the cases where only bi-grams (B), or all *N*-grams (M) are considered. False negative ($\textrm{BF}<1$ under $H_1$) and false positive ($\textrm{BF}>1$ under $H_2$) results are highlighted in bold. The misclassification rate is reported in the last row.

BF	$H_1$ (*Human*)				$H_2$ (*ChatGPT*)			
	FSI		Students		FSI		Students	
	B	M	B	M	B	M	B	M
$10^{-8}-10^{-7}$	0	0	0	0	0	0	0	2
$10^{-7}-10^{-6}$	0	0	0	0	0	2	0	6
$10^{-6}-10^{-5}$	0	0	0	0	0	7	0	14
$10^{-5}-10^{-4}$	0	0	0	0	0	9	0	8
$10^{-4}-10^{-3}$	0	0	0	0	0	5	0	9
$10^{-3}-10^{-2}$	0	0	0	0	0	9	0	6
$10^{-2}-10^{-1}$	**1**	0	0	0	22	2	26	3
$10^{-1}-1$	**5**	**2**	**10**	0	13	2	16	0
$1-10$	15	1	22	3	**2**	0	**7**	**1**
$10-10^{2}$	18	3	22	1	0	**1**	0	0
$10^{2}-10^{3}$	16	4	18	6	0	0	0	0
$10^{3}-10^{4}$	2	4	0	6	0	0	0	0
$10^{4}-10^{5}$	0	8	0	9	0	0	0	0
$10^{5}-10^{6}$	0	7	0	20	0	0	0	0
$10^{6}-10^{7}$	0	11	0	11	0	0	0	0
$10^{7}-10^{8}$	0	4	0	9	0	0	0	0
$10^{8}-10^{9}$	0	6	0	3	0	0	0	0
$10^{9}-10^{10}$	0	7	0	4	0	0	0	0
$\mathrm{BF>1}$	51	55	62	72	**2**	**1**	**7**	**1**
$\mathrm{BF<1}$	**6**	**2**	**10**	0	35	36	42	48
Misclassification rate	0.10	0.03	0.14	0.00	0.05	0.03	0.14	0.02

Performances are very promising with a small number of false negatives and false positives. There are some key aspects that must be raised. The Bayes factors giving rise to false results have a modest magnitude. They are, in fact, mainly located in the interval ($10^{-1}-1$) whenever $H_1$ is true and a value greater than 1 is expected, and in the interval ($1-10$) whenever $H_2$ is true and a value lower than 1 is expected. The support provided by values of this magnitude is generally considered weak. The term ‘weak’ refers to a six-point verbal scale for values of the BF greater (lower) than 1 with six adjectives for hypothesis support of weak, moderate, moderately strong, strong, very strong and extremely strong and corresponding numerical ranges for the BF^17^. The best performance is obtained with the multivariate model (M), which takes into account the complexity of text styles to an extent that cannot be captured by a single variable.

**Figure 2:**
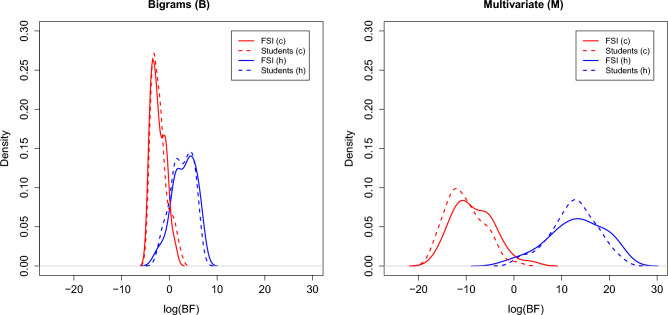
Weights of evidence, log(BF), for every texts written by (1) ChatGPT on forensic (solid red-colored line) and socio-psycho-artistic themes (dashed red-colored line) and by (2) humans scientists (solid blue-colored line) and students (dashed blue-colored line).

Figure 2 is of extreme interest. On the one hand, there are traced graphically (in red color) the densities’ estimates from BF values (on logarithmic scale) obtained once evaluating texts delivered by ChatGPT. A distinction is made between scientific articles (FSI, solid curve), and students’ texts (dashed curve). On the other hand (in blue color), one may observe the densities’ estimates from BF values (on logarithmic scale) obtained once evaluating texts written by human beings. It should be noted that similar performance was also obtained when considering databases of smaller sample sizes. This is a key aspect supporting the suitability of the model for author discrimination, which meets the needs encountered in practice when large databases are not necessarily available.

Results summarized in Figure 2 allows us to draw two unprecedented considerations: (1) the nearly superposition of the distributions of BF values (solid and dashed curves) highlights an absence in terms of topic influence between texts written by humans or ChatGPT whenever such measure of redundancy is employed. This means that a scientific forensic text is not distinguished from texts on socio-psycho-artistic topics. Furthermore, (2) the superposition also supports the hypothesis that the factor ‘language’ (text written in English or French) does not influence the results. The results obtained are in agreement with those of other studies conducted in this field (see the review provided by^18^).

There is a last aspect that should be tackled. The classification task can in fact be formulated as a decision problem, with $d_{1(2)}$ representing the decision of classifying a questioned text as written by a human being (ChatGPT), while $l_{1(2)}$ represents the loss that is incurred whenever decision $d_{1(2)}$ is incorrect (i.e., decision $d_{1(2)}$ is taken and hypothesis $H_{1(2)}$ is not true).

The formal Bayesian decision criterion is to calculate the *expected loss* for each decision, and decide $d_1$ (i.e., classify the questioned text as written by a human writer) if it gives rise to a smaller expected loss. This represents the coherent classification procedure since it minimizes the probability of misclassification (see, e.g.^19–21^). Whenever there are assessed equal prior probabilities for the hypotheses of interest ($\Pr (H_1)=\Pr (H_2)$), and a symmetric loss function ($l_1=l_2$) is chosen (that is, it is felt that adverse decision outcomes are equally undesirable) this amounts to decide $d_1$ ($d_2$) whenever the BF is greater (smaller) than 1^22^.

It might be questioned that a symmetric loss function could not necessarily represent a coherent choice for this context. In fact, one may agree that falsely classifying a text as written by the artificial intelligence should be regarded more severely than falsely classifying a text as written by a human. Therefore, $l_2$ may be taken larger than $l_1$ (e.g. $l_2=10 l_1$, meaning that falsely classifying a text as written by AI is considered ten times as serious as the opposite). The assignment of an asymmetric loss function, as well as non equal prior probabilities concerning the competing hypotheses, may sensibly alter the classification threshold and consequently the decision on authorship, with a signficant decrease in the misclassification rate.

### An example under constraint of limited sample size

It should be emphasized that forensic practice is routinely faced to limited amount of trace material and comparative reference samples. It may be therefore worth investigating how the proposed probabilistic approach performs in this situation. For this reason, a scenario characterized by a poor amount of available material has been considered and analysed.

Consider a written text whose authorship is questioned and suppose it is of interest to discriminate between the following two hypotheses: $H_1$, the questioned text has been written by the economic Nobel prize winner Paul Krugman *versus* $H_2$, the questioned text has been generated by ChatGPT. Five texts on economics subjects written by Paul Krugman and five texts on a same economics content, generated by ChatGPT, were used to define the two populations.

The Paul Krugman’s texts - cut at 3100 characters - are the following:

1. Paul Krugman, Is This the End of Peace Through Trade? *New York Times*, December 13, 2022;
2. Paul Krugman, Learning From the Southwest Airlines Fiasco, *New York Times*, December 29, 2022;
3. Paul Krugman, The Football Game Theory of Inflation, *New York Times*, January 3, 2022;
4. Paul Krugman, Election Deniers Are Also Economy Deniers, *New York Times*, January 9, 2022;
5. Paul Krugman, The G.O.P.’s Long War Against Medicare and Social Security, *New York Times*, January 13, 2022.

This *corpus* has been collected and analyzed by^23^, who obtained the necessary redundancy measures starting from sequences of N-grams (i.e. based on words).

ChatGPT texts were generated using a prompt similar to the one described in section ‘Available material’.

Every text has been analysed using the redundancy measure for uni-, bi-, tri- and quadri-grams. Then, in turn, every text authored by Paul Krugman and ChatGPT, respectively, has been taken as evidence and tested under the two hypotheses $H_1$ and $H_2$, and the Bayes factor has been calculated as in (1). Bayes factor results have been obtained following this leave-one-out procedure. Results for bi-grams are reported in Table 2. It can be observed that no error is reported, as the Bayes factors always support the correct hypothesis. In fact, the BFs obtained under hypothesis $H_1$ are always greater than 1, while the BFs obtained under hypothesis $H_2$ are always smaller than 1. Note that analogous performance is obtained for other *N*-grams.

**Table 2:** Assessment of the performances of the proposed probabilistic approach for authorship discrimination when the putative source is Paul Krugman ($H_1$), and when the putative source is ChatGPT ($H_2$). Analyses results are reported for the cases where only bi-grams (B) are considered. No false negativse ($\textrm{BF}<1$ under $H_1$) or false positives ($\textrm{BF}>1$ under $H_2$) are observed.

BF	$H_1$ (*Krugman*)	$H_2$ (*ChatGPT*)
$<10^{10}$	0	0
$10^{-10}-10^{-5}$	0	2
$10^{-5}-10^{-4}$	0	0
$10^{-4}-10^{-3}$	0	1
$10^{-3}-10^{-2}$	0	0
$10^{-2}-10^{-1}$	0	2
$10^{-1}-1$	0	0
$1-10$	3	0
$10-10^{2}$	2	0
$>10^{2}$	0	0
$\textrm{BF}>1$	5	0
$\textrm{BF}<1$	0	5

## Discussion and conclusion

This paper takes advantage of an original measure, the redundancy value, for authorship purposes, while conventional approaches typically rely on machine learning (see, e.g.,^24^). A Bayesian probabilistic approach was proposed and its performance was analyzed for various *N*-grams in a uni- and multi-variate format. The multivariate format offers the best classification rate results to discriminate between human and ChatGPT texts. Despite a limited population size, the obtained results are of great interest as they’re topic- and language-independent.

An alternative scenario characterized by a small amount of background information has also been analyzed in order to study the performance of the proposed probabilistic approach under such extreme condition, which may nevertheless correspond to the daily work of a forensic expert.

Though for operational purposes, a wider reference sample may be preferable, results reported in this paper provide solid arguments in support of the view that stylometry and a probabilistic approach offer a promising framework to successfully address emerging investigative issues concerning questioned authorship that the widespread use of artificial intelligence is making more and more challenging.

## Data Availability

The data that support the findings of this study are available on request from the corresponding author.
